# Detecting Water Diversion Fingerprints in the Danjiangkou Reservoir from Satellite Gravimetry and Altimetry Data

**DOI:** 10.3390/s19163510

**Published:** 2019-08-10

**Authors:** Nengfang Chao, Gang Chen, Zhicai Luo, Xiaoli Su, Zhengtao Wang, Fupeng Li

**Affiliations:** 1 College of Marine Science and Technology, China University of Geosciences, Wuhan 430074, China; 2 Hubei Subsurface Multi-Scale Imaging Key Laboratory, Institute of Geophysics and Geomatics, China University of Geosciences, Wuhan 430074, China; 3 MOE Key Laboratory of Fundamental Physical Quantities Measurement, Hubei Key Laboratory of Gravitation and Quantum Physics, School of Physics, Huazhong University of Science and Technology, Wuhan 430074, China; 4 Key Laboratory of Geospace Environment and Geodesy, School of Geodesy and Geomatics, Wuhan University, Wuhan 430079, China

**Keywords:** GRACE, ILMM, altimetry, water diversion fingerprints, Danjiangkou Reservoir

## Abstract

The Danjiangkou Reservoir (DJKR) is the freshwater source for the Middle Route of the South-to-North Water Diversion Project in China, and its water level and storage changes are important for water resource management. To maximize the potential capacity of the Gravity Recovery and Climate Experiment (GRACE) mission, an improved Lagrange multiplier method (ILMM) is first proposed to detect terrestrial water storage anomalies (TWSA) in the small-scale basin (DJKR). Moreover, for the first time, water diversion fingerprints are proposed to analyze the spatiotemporal pattern of the TWSA in the DJKR. The results indicate that the increased water level and storage signals due to the DJKR impoundment in 2014 can be effectively detected by using the ILMM, and they agree well with the results from altimetry and in situ data. Additionally, the water diversion fingerprints due to the DJKR impoundment are inferred, and describe the progression of spatiotemporal variability in water storage. The results show that water storage decreased in the upper Hanjiang River and increased in the DJKR as well as to the east of it during the period 2013–2015. Our research provides a scientific decision-making basis for monitoring the water resources of the DJKR and managing the South-to-North Water Diversion Project.

## 1. Introduction

Surface water that is stored in lakes, reservoirs, and rivers plays a large role in agricultural irrigation, aquaculture, hydroelectric power, disaster prevention and mitigation, human life, and industrial activities [1]. However, surface water resources are unevenly distributed in China, with abundant freshwater in the south and water scarcity in the north. To address this issue, the Chinese government decided to implement the South-to-North Water Diversion Project (SNWDP) in 2002, which includes three routes: East, Middle, and West. In particular, the Middle Route of the SNWDP (MRSNWDP) was devised to divert freshwater from the Danjiangkou Reservoir (DJKR) to Henan and Hebei provinces and the Beijing and Tianjin municipalities in China [2]. The Hanjiang River is the longest tributary of the Yangtze River [3], and approximately 70% of the freshwater is diverted to the DJKR. The DJKR is the freshwater source of the MRSNWDP. The water level and storage changes in the DJKR are greatly important for managing the water resources of the SNWDP.

Changes in water resources are strongly related to the sustainable development of society and the economy, and they are generating wide public concern. One main tactic used by humans to control water resources is the construction of dams or reservoirs. Human-induced changes in water resources contribute to sea level variation in two major ways: a positive contribution is the loss of water due to excessive groundwater depletion, and a negative contribution is the gain of water because of artificial reservoir impoundment [4]. Deriving the change in water storage in artificial reservoirs can help us precisely quantify the human influence on the water system [5]. If the amount of impounded water in artificial reservoirs can be precisely determined, it can also benefit our understanding of the global water distribution and global sea-level variables [6].

Currently, artificial reservoirs are monitored with considerable effort devoted to obtaining an accurate estimation of the available water resources. In situ hydrological gauges can provide precise observational data, but their consumption is large and hard to maintain. The number of operational gauges has rapidly decreased, and as a result, limited data are available for hydrological research, particularly for studies on the hydrological cycle [7,8]. Therefore, there is an urgent need to develop other methods to obtain hydrological data.

One alternative method is satellite altimetry. Satellite altimetry involves determining surface heights by measuring the two-way travel time of an electromagnetic pulse between the altimeter and the surface, and is used to monitor water-level changes from space [9,10]. The advantages of this method are the lack of additional costs and the provision of global observational data. Satellite altimetry is a successful technique that has been widely used to monitor variations in lake, reservoir, and river levels [11]. The comprehensive monitoring of surface resources not only requires knowing the water-level changes, but also requires knowledge of the extent of the surface water and water volume. Here, multi-source satellite altimetry missions and satellite images from Landsat 7 are used to determine the surface water storage change in the DJKR.

Another effective method is satellite gravimetry. Artificial reservoirs are important indicators of human influences on the environments, and their cumulative impacts on regional water storage cause the gravity signal to change, which can be potentially detected by satellite gravimetry. Since the Gravity Recovery and Climate Experiment (GRACE) mission [12] was successfully launched in March 2002, it has been comprehensively used for monitoring terrestrial water storage anomalies (TWSA). It has considerably promoted the development of hydrology research. TWSA include soil moisture, surface water bodies (lakes, rivers, and reservoirs), groundwater, glaciers, snow water equivalence, and canopy water storage [6]. TWSA determined from GRACE data (GRACE-TWSA) have been utilized for various hydrological applications, such as monitoring floods [13,14], drought [15,16], groundwater [17], and surface reservoir storage changes [18].

Due to the limitation of the GRACE resolution (~3° × 3°), most applications have involved large-scale regions, and few studies have been devoted to small-scale regions, such as artificial reservoirs. The results from Lorenz et al. [19] emphasized that the spatial resolution of the GRACE mission was not the only critical factor for studying water resources. They cited the signal strength as another determinant, and termed it the ‘gravimetric resolution’. The latest results from Yi et al. [20] showed that the signal of a small (~0.2° × 0.2°) reservoir can potentially be detected by GRACE data if the mass change is larger than 6 Gt. In the study presented herein, an improved Lagrange multiplier method (ILMM) is proposed to detect the TWSA in a small-scale basin (DJKR).

Several studies have investigated sea-level fingerprints (SLF) [21]. SLF are the characteristic signatures of sea level changes due to a specific mass source [22], and they are used to describe the interaction between sea level changes and solid earth deformation coupled with gravitation. In the current study, similar to the concept of SLFs, water diversion fingerprints in the land (DJKR) are quantified for the first time. Water diversion fingerprints are derived by using data from GRACE, multi-source altimetry missions, Landsat, land surface models, precipitation, and in situ measurements.

The main contributions and novelties of this research are as follows: (1) the relationship between changes in water level from in situ observations and the areas from Landsat images is determined in the DJKR; (2) a new method of inferred leakage correlation and ILMM is proposed to detect the TWSA in the DJKR; (3) the signal from the DJKR impoundment in 2014 is captured by using GRACE and multi-source altimetry missions for the first time; and (4) the water diversion fingerprints due to DJKR impoundment are derived and verified for the first time.

## 2. Materials and Methods

### 2.1. Study Area

**Figure 1:**
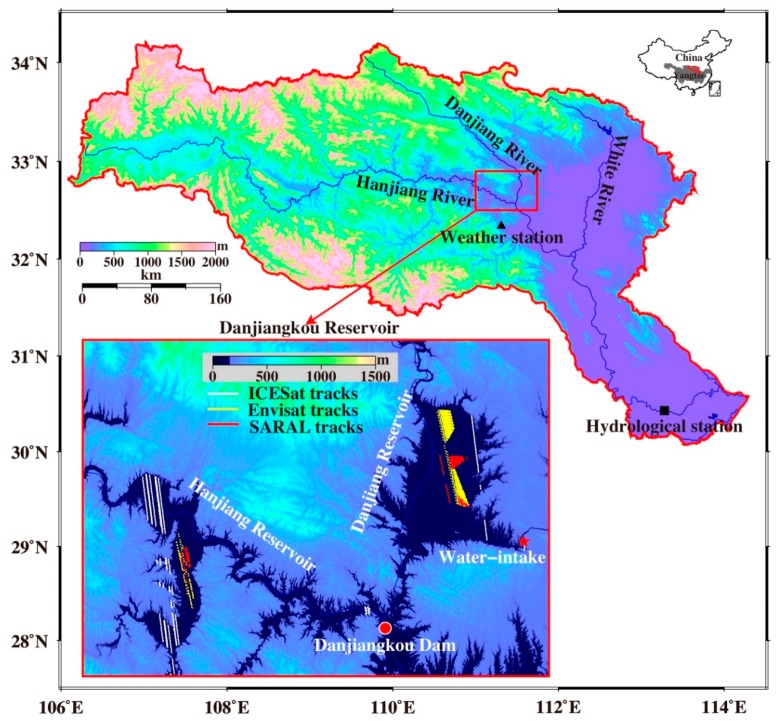
The location of the study area (Danjiangkou Reservoir, or DJKR: the red rectangular region), the distribution of in situ hydroclimatic gauges, and the altimetry mission tracks in the DJKR water bodies (the red rectangular region). The red solid surface marks the Hanjiang River basin (HRB), the triangle marks the Danjiangkou weather station, the square marks the Xiantao hydrological station, the circle marks the Danjiangkou Dam and in situ water level, and the five-pointed star marks the freshwater supply for the Middle route of the South-to-North Water Diversion Project (SNWDP).

The DJKR (32°36′–33°48′ N, 110°59′–111°49′ E) is located upstream of the Hanjiang River at the junction between the Hanjiang River and Danjiang River and forms a “V” shape. It includes the Hanjiang and Danjiang Reservoir areas, and distributes water to Hubei and Henan provinces in China (Figure 1). With a total drainage area of approximately 95,000 km^2^ and an average water storage volume of approximately 39.48 billion cubic meters, the DJKR is the largest artificial freshwater lake in Asia [23]. In addition, the DJKR is a top-grade multi-purpose reservoir with flood control, electricity generation, navigation, and agricultural irrigation functions. In this study, data from altimetry missions were used to monitor the water level change in the DJKR (the red rectangular region in Figure 1), and GRACE mission data were used to detect the TWSA in the total drainage basin.

The location of the DJKR has a subtropical monsoon climate, which has remarkable transitional climatic characteristics. The annual mean temperature and precipitation are 14.4–15.7 °C and 800–1000 mm, respectively, and 80% of the rainfall occurs between May and October [24]. The upper Hanjiang River is the source of 70% of the runoff of the DJKR, and the remaining 30% comes from the Danjiang River, which is the longest tributary of the Hanjiang River. The DJKR has a complex topography, with mountains and hills forming approximately 97% of the area.

To implement the MRSNWDP, the height of the Danjiangkou Dam (DJKD) was increased from 162.0 m to 176.7 m, and its normal water level and storage capacity were increased from 157.0 m to 170.0 m and from 1.7 × 10^10^ m^3^ to 2.9 × 10^10^ m^3^, respectively [25]. The DJKR was impounded in October 2013, and began diverting water to Beijing, Tianjin, Henan, and Hebei in December 2014.

### 2.2. Data

The area changes in the DJKR were determined by using Landsat 7 images, and the TWSA were detected by using GRACE data. The Global Land Data Assimilation System (GLDAS) [26], Climate Prediction Center (CPC) [27], WaterGAP Global Hydrology Model (WGHM) [28], and Community Land Model (CLM4.0) [29] were used to determine the scale factors [30]. Altimetry data were used to determine the water-level change in the DJKR, and in situ water-level data were used to establish the relationship with the area of the DJKR and verify the water-level changes identified from altimetry data and the TWSA detected from GRACE data. In addition, precipitation, evapotranspiration, GLDAS, and CLM4.0 were used to support the GRACE-TWSA-based detection of water diversion fingerprints in the DJKR.

#### 2.2.1. Satellite Images

Data from the Thematic Mapper (TM), Enhanced Thematic Mapper Plus (ETM+), Operational Land Imager (OLI), and Moderate Resolution Imaging Spectroradiometer (MODIS) were successfully applied to estimate the area of the lakes, reservoirs, and rivers [31]. Although MODIS data a have high temporal resolution, their spatial resolution is approximately 250–1000 m, and the narrowest width of the DJKR is approximately 300 m. The spatial and temporal resolutions of Landsat multispectral images (TM, ETM+, and OLI) are 30 m and 16 days, respectively. Landsat spectral bands 1–7 were used. Here, Landsat 7 multispectral images (path/row 138/037, TM and OLI) captured during the period of 2011–2014 were collected from the United States (U.S.) Geological Survey (USGS) server [32].

#### 2.2.2. GRACE Data

The ILMM requires an error in the satellite measurement, which is the formal error (Wahr et al., 2006) of the GRACE-variable gravity field model. It was not provided by the Center for Space Research (CSR) [33,34]. To compare with the results of the MASCON from the Jet Propulsion Laboratory (JPL), GRACE data were obtained from the JPL Release 06 from April 2002 to March 2016. The $C_{20}$ term of the GRACE time-variable gravity field model was determined from satellite laser ranging observational data [35]; the degree-one harmonic coefficients (Earth’s geocenter) were estimated from Swenson et al. [36], and correction for the glacial isostatic adjustment (GIA) was done following A. Geruo et al. [37].

TWSA were also obtained from the GRGS (Groupe de Recherche de Géodésie Spatiale, etc. [38]) with a degree and order of 80, which applied DDK5 filtering [39] to the data from the CSR, GeoForschungsZentrum Postdam (GFZ), and JPL. The method used to estimate the TWSA from the above products is described in Wahr et al. [40]. The results from the JPL fan-shaped (FAN) filter [41], JPL mass concentration (MASCON) [42], and CSR MASCON [43] were also compared.

#### 2.2.3. Altimetry Data

##### Laser Altimetry Data

The Ice, Cloud, and land Elevation Satellite (ICESat) mission, which is part of the National Aeronautics and Space Administration (NASA) Earth Observing System (EOS), was launched in January 2003 from Vandenberg Air Force Base; it ended in August 2010. The sole instrument on board ICESat was the Geoscience Laser Altimeter System (GLAS), which is a laser altimeter that provides a high-precision dataset. As the ICESat orbited, the GLAS produced a series of laser spots of approximately 70 m in diameter that were separated by nearly 170 m along the spacecraft ground track [44]. From 2003 to 2009, ICESat/GLAS observed 19 campaigns with a ground track cycle of 8 or 91 days. Here, the data from GLA14 (Version 34) [45] were used.

##### Radar Altimetry Data

In this study, we used the 20-Hz Environmental Satellite (Envisat) Geophysical Data Record (GDR) data product in version 2.1 [46] and the 40 Hz Satellite with ARgos and ALtiKa (SARAL) GDR data product in the Calibration/Validation (Cal/Val) phase. Since the quality of the Envisat data might not be good at the mission-commissioning phase, this study applied data that were obtained between January 2003 and September 2010 with a repeat cycle of 35 days. The SARAL reached the historical orbit of Envisat at the end of October 2013 [47] and transitioned to a drifting orbit in July 2016. Thus, for the Ka band, we chose altimetry data that were collected between November 2013 and March 2016 with a repeat cycle of 35 days.

#### 2.2.4. Model Data

(1) GLDAS: The Global Land Data Assimilation System (GLDAS) [26] includes four monthly land surface models: the CLM (the Community Land Model), Mosaic (MOS), VIC (the Variable Infiltration Capacity), and NOAH (National Centers for Environmental Prediction/Oregon State University/Air Force/Hydrologic Research Lab Model), which do not include surface water storage in lakes, reservoirs, and river channels. The average of the four monthly land surface models with soil moisture states in a 1° × 1° grid was used to estimate the water storage in the top 2 m of the soil layer. GLDAS include soil moisture, snow, and vegetation canopy storage, excluding surface water storage in lakes, reservoirs, and river channels. Therefore, GLDAS was subtracted by the GRACE data to infer the total change in surface water storage.

(2) CPC: The Climate Prediction Center (CPC) [27] with soil moisture states in 0.5° × 0.5° with monthly time steps were used to estimate the water storage.

(3) WGHM: The WaterGAP Global Hydrology Model (WGHM) 2.2 [28], with a spatial resolution of 0.5° in monthly time steps, was used to estimate TWSA.

(4) CLM4.0: The National Center for Atmospheric Research (NCAR) Community Land Model (CLM4.0) [29] simulates the partitioning of mass and energy from the atmosphere, the redistribution of mass and energy within the land surface, and the export of freshwater and heat to the oceans. The spatial and temporal resolutions of CLM4.0 are 0.9° × 1.25° and monthly, respectively. Components of terrestrial water storage output by the CLM4.0 include soil moisture, snow, vegetation canopy storage, channel storage in rivers, and climate-driven change; human activities are excluded. GRACE-TWSA is the total water storage change. Therefore, human-induced TWSA can be determined by GRACE subtracted by CLM4.0.

#### 2.2.5. Precipitation, Evapotranspiration, and Water-Level Data

The monthly precipitation data are from the Tropical Rainfall Measuring Mission (TRMM), which has spatial and temporal resolutions of 0.25° × 0.25° and monthly, respectively [48], as well as the Danjiangkou weather station (Danjiangkou, China) (China Meteorological Data Service Center [49]) and the Xiaotao hydrological station (Xiantao, China) (Chang Jiang Water Resources Commission of the Ministry of Water Resources [50]). Here, evapotranspiration data are from different products, i.e., monthly evapotranspiration products from GLDAS, Moderate Resolution Imaging Spectroradiometer (MODIS), and the Danjiangkou weather station. The GLDAS data integrate satellite data and Land Surface Model (LSM) data to generate a global distribution of land surface states (e.g., evapotranspiration). Evapotranspiration data from MODIS with spatial and temporal resolutions of 1° × 1° and monthly are based on the Penman–Monteith method, in which remote sensing data and meteorological observations are combined [51]. The in situ water level data at the DJKD observation station are from the Hydrology and Water Resources Survey Bureau of China [52].

### 2.3. Methods

#### 2.3.1. Deriving the DJKR Area Change from Landsat 7

The steps for capturing the area of the DJKR from Landsat 7 images are as follows: (1) preprocess the original images via radiation correction, geometric correction, and atmospheric correction [3], transform them to the Universal Transverse Mercator (UTM) projection, and obtain the reflectance data; (2) extract the water body information by using the modified normalized difference water index (MNDWI) [53]; (3) determine the threshold segmentation by using the Otsu method [54]; and (4) remove the water data that are outside of the reservoir, count the total number of pixels, multiply by the area of the pixels, and obtain the area of the DJKR as the final output.

Changes in water volume (i.e., changes in mass) cannot be estimated by only using water-level data. Thus, the relationship between the change in area and water level should be obtained. The most widely used method is to combine the area retrieved from satellite images and the water level from multi-source altimetry missions or from in situ observations.

#### 2.3.2. Estimating the Water-Level Change in the DJKR from Altimetry Data

In Frappart et al.’s study for European ENVIronmental SATellite (ENVISAT) validation over the Amazon basin [55], the elevation change retrieved by the ice-1 algorithm [56] agreed best with in situ gauge data from the inland water level. Here, the altimetry data (ENVISAT and SARAL) were retrieved by the ice-1 algorithm [57] to perform further analysis. To retrieve the elevation change in the water level in the DJKR, geophysical range corrections, including (European Centre for Medium-Range Weather Forecasts) modeled wet and dry troposphere delays, the ionosphere delay, solid Earth tides, and pole tides were applied [56]. Due to the potential contamination by radar echoes from land reflectors and the locking loss of onboard trackers near the coasts, only data near the center of the lake were used. The Envisat and SARAL tracks are shown in Figure 1.

The ICESat laser altimeter measured elevations in 18 cycles from 2003 to 2009, with each cycle lasting 12–15 days. Previous studies, such as that by Nicolas et al. [58], have shown that the mean accuracy of the ICESat-derived elevations over flat deserts is ±15 cm. Therefore, ICESat has sufficient accuracy to determine changes in the lake and reservoir water levels. ICESat observations were edited using the following empirical procedure: (1) remove the ICESat elevations that exceed the minimum and maximum Shuttle Radar Terrestrial Mission (SRTM) elevations within the study region; (2) remove the elevations at two consecutive along-track footprints that have a difference exceeding 15 m (the rationale for this approach is that allowing a maximum lake/reservoir slope of 5° for two neighboring along-track footprints spaced at approximately 172 m results in a maximum elevation difference of approximately 15 m); and (3) determine a smoothed value $\bar{h_{n}}$ at a given point using n neighboring points (n = 50 in this study). Along a sufficiently long ground track, the differences between the original and smoothed heights were computed to determine the standard deviation ($\delta_{n}$). Then, if the original height ($h_{i}$) fit the condition $\left|h_{i}-\bar{h_{n}}\right|>3\delta_{n}$, then $h_{i}$ was removed. The final ICESat tracks are shown in Figure 1. Moreover, the biases between the different altimetry missions (ENVISAT, SARAL, and ICESat) were corrected by eliminating systematic errors, and the surface water-level results from different altimetry missions in different places were the regional average values in the DJKR water bodies.

#### 2.3.3. Improved Lagrange Multiplier Method to Infer TWSA in the DJKR

Due to the flight characteristic factors of the GRACE task and the limits of the background and current mathematical models, the GRACE-TWSA must be filtered to eliminate the north–south striping error [40]:

(1) $$TWSA(\theta ,\lambda )=\frac{a\rho_{\mathrm{ave}}}{3\rho_{w}}\sum_{l=0}^{L}\frac{2l+1}{1+k_{l}}W_{l}\sum_{m=0}^{l}{\bar{P}}_{lm}(\sin\theta )[(\Delta C_{lm}\cos m\lambda +\Delta S_{lm}\sin m\lambda )]$$

where *λ* is the geocenter longitude; *θ* is the geocenter latitude; *a* is the semi-major axis of the reference ellipsoid; *l*, *m* are the degree and order; *L* is the highest degree; $\rho_{ave}$ is the average Earth density (5517 kg/m^3^); $\rho_{w}$ is the water density (1000 kg/m^3^); $k_{l}$ is the Love number [59]; ${\bar{P}}_{lm}$ is the completely normalized Legendre association function [60]; $\Delta C_{lm}$ and $\Delta S_{lm}$ are the residual spherical harmonic coefficient after subtracting the long-term mean of Earth’s gravity field, which is about the normal Earth gravity field; and $W_{l}$ is the smoothing coefficients of the Gaussian filter [40,61].

Smoothing methods can also cause signal attenuation and leakage. Therefore, the scale factor (*S*), leakage ($l_{}^{m}$), and bias ($b_{}^{m}$) were obtained on the basis of the hydrological models from Landerer and Swenson [30], Longuevergne et al. [62], and Klees et al. [63]:

(2) $$s\to \min\left\{M(\theta ,\lambda )-s\bar{M}(\theta ,\lambda )\right\}$$

(3) $$l_{}^{m}=\frac{1}{A_{}}\int_{\Omega }M(\theta ,\lambda )R^{*}(\theta ,\lambda )\bar{R}(\theta ,\lambda )d\Omega$$

(4) $$b_{}^{m}=\frac{1}{A}\int_{\Omega }M(\theta ,\lambda )(R(\theta ,\lambda )-\bar{R}(\theta ,\lambda ))d\Omega$$

where *θ* is the colatitude, *λ* is the geocentric longitude, dΩ=sinθdθdλ, M(θ,λ) is the value from the models, and R(θ,λ) is a function of the study region:

(5) $$R(\theta ,\lambda )=\{\begin{array}{cc} 1, & \mathrm{inside}\;\mathrm{study}\;\mathrm{area}\;\Omega \\ 0, & \mathrm{elsewhere} \end{array}$$

A bar over M(θ,λ) and R(θ,λ) indicates their first filter.

To determine the leakage, the outside region of the study area is defined as:

(6) $$R^{*}(\theta ,\lambda )=1-R(\theta ,\lambda )=\{\begin{array}{cc} 0, & \mathrm{inside}\;\mathrm{study}\;\mathrm{area}\;\Omega \\ 1, & \mathrm{elsewhere} \end{array}$$

$A^{*}=\int_{\Omega }R{\left(\theta ,\lambda \right)}^{*}d\Omega =4\pi -A$, $A=\int_{\Omega }R(\theta ,\lambda )d\Omega$.

The above methods are highly dependent on the hydrological model, which can cause scaling factors to differ considerably, especially in semi-arid, arid, and over-irrigated areas [64]. GRACE-TWSA can be considered a hydrological signal, but this signal differs from hydrological models. If the two types of data are fitted, signal aliasing can occur. Therefore, a scale factor that is independent of the hydrological model was proposed by Dutt Vishwakarma et al. [65], so it is called the DV method (DVM) herein:

(7) $$s=\frac{\int_{\Omega }R(\theta ,\lambda )d\Omega }{\int_{\Omega }R(\theta ,\lambda )\bar{R}(\theta ,\lambda )d\Omega }$$

The TWSA (Equation (1)) in the spatial domain can be expressed as:

(8) $$f(\theta ,\lambda )=f(\theta ,\lambda )R(\theta ,\lambda )+f(\theta ,\lambda )R^{*}(\theta ,\lambda )=F(\theta ,\lambda )+F^{*}(\theta ,\lambda )$$

The first filter is:

(9) $$\begin{array}{ll} \bar{f}(\theta ,\lambda ) & =\frac{1}{A}\int_{O}f(\theta ,\lambda )\bar{R}(\theta ,\lambda )d\Omega \\ & =\frac{1}{4\pi }\int_{\Omega^{\prime }}\left(F(\theta ,\lambda )+F^{*}(\theta ,\lambda )\right)b(\theta ,\lambda ,\theta^{\prime },\lambda^{\prime })d\Omega^{\prime }\\ & =\bar{F}(\theta ,\lambda )+{\bar{F}}^{*}(\theta ,\lambda ) \end{array}$$

The abbreviation expression is:

(10) $$\bar{f}=\bar{F}+l$$

where *l* is the leakage and is calculated by:

(11) $$l=\frac{1}{A}\int_{\Omega }f(\theta ,\lambda )R^{*}(\theta ,\lambda )\bar{R}(\theta ,\lambda )d\Omega$$

With $f=s\bar{F}$:

(12) $$\int_{\Omega }f(\theta ,\lambda )R(\theta ,\lambda )d\Omega =s\cdot \int_{\Omega }\bar{F}(\theta ,\lambda )R(\theta ,\lambda )d\Omega$$

Thus, the leakage from Equation (11) can be transformed by using Equation (7):

(13) $$\begin{array}{ll} l & =\frac{1}{A}\int_{\Omega }f(\theta ,\lambda )R^{*}(\theta ,\lambda )\bar{R}(\theta ,\lambda )d\Omega =\frac{1}{A}\int_{\Omega }f(\theta ,\lambda )R(\theta ,\lambda ){\bar{R}}^{*}(\theta ,\lambda )d\Omega \\ & =\frac{s}{A}\int_{\Omega }\bar{F}(\theta ,\lambda )R(\theta ,\lambda ){\bar{R}}^{*}(\theta ,\lambda )d\Omega \end{array}$$

Here, we used $f=s\bar{F}$ to derive a new expression of leakage *l* (Equation (13)).

Therefore, from Equations (7), (10), and (13), we can determine the surface mass change expression, which does not need the hydrological model to restore the signal and correct the leakage:

(14) $$\bar{f}=\frac{f}{s}+l\Leftrightarrow f=s(\bar{f}-l)$$

According to Equations (7), (9), (13), and (14), the key to improving the accuracy of mass change is the determination of the optimal smooth kernel for the study area.

To estimate the regional surface mass changes using GRACE data, Swenson et al. [66] proposed the Lagrange multiplier method (LMM), which reduced the impact of GRACE observation errors. The key step in this method is the determination of the Lagrange parameter *λ*, which is obtained by the minimum satellite measurement error and signal leakage error, and only the formal error is needed.

The LMM is a spatial smoothing method [66] that requires the restoration and correction of the signal. Here, in the improved LMM (ILMM), the signals were restored and corrected after applying the LMM. The process of the ILMM comprises the following steps: (1) the TWSA are first determined by using the LMM; (2) the restored signal and corrected signal are determined by using Equations (7) and (13); and (3) the TWSA results are finally determined from Equation (14). More details on the Lagrange multiplier method are in Appendix A, Swenson et al. [66], and Chao et al. [14].

From the above descriptions, the surface mass change can be inferred by different methods.

(1) Scale factor method (SFM) [30]:
  (15) $$f=s\bar{f},\;s\to \min\left\{M(\theta ,\lambda )-s\bar{M}(\theta ,\lambda )\right\}$$
(2) Additive approach (AA) [63]:
  (16) $$f=\bar{f}-l_{}^{m}+b_{}^{m}$$
(3) Multiplicative approach (MA) [62]:
  (17) $$f_{}=s(\bar{f}-l_{}^{m})$$
(4) The LMM [66];
(5) The ILMM (this study), which is based on the LMM and Equations (7), (13), and (14).

#### 2.3.4. Inferring Total Surface Water Storage and Human-Induced Surface Water Storage Anomaly

The total TWSA from GRACE can be disaggregated into soil moisture anomaly (SMA), surface water storage anomaly (SWSA), and groundwater storage anomaly (GWSA) [6]:

(18) TWSA=SMA+SWSA+GWSA

Here, our study area is the DJKR; the total surface water storage anomaly (TSWSA) is defined as the sum of the SWSA and GWSA, and the human-induced surface water storage anomaly (HSWSA) can be represented by the difference between the TSWSA and climate-driven surface water storage anomaly (CSWSA). Arranging Equation (18) [67,68]:

(19) $$\begin{array}{c} TSWSA=SWSA+GWSA=TWSA-SMA\\ HSWSA=TSWSA-CSWSA=TWSA-SMA-CSWSA \end{array}$$

SMA can be obtained from GLDAS or CLM4.0, and CSWSA can be obtained from CLM4.0 [68,69]. Here, TSWSA is determined by combining GRACE and GLDAS, and CSWSA is determined by combining GRACE and CLM4.0, which is the same as approach as that in the studies of Voss et al. [67] and Joodaki et al. [68].

## 3. Results

### 3.1. The Area Change in the DJKR from Landsat

**Figure 2:**
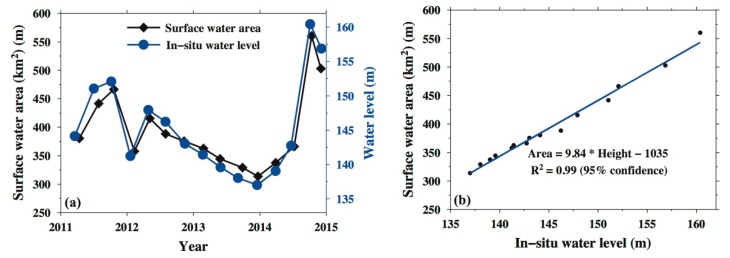
(**a**) Changes in the area and water levels in the DJKR (the red rectangular region in Figure 1); (**b**) linear regression model between the change in the area and water level.

The surface area of the DJKR expansion or reduction responds to a water level increase or decrease, respectively, which is proven in Figure 2a. The surface area and water-level change can be affected by climate variability and human activities. The natural (climate-driven) variability is mainly from climate-derived precipitation and evapotranspiration changes, which result in seasonal changes in surface area and water level. Human-induced changes include irrigation, drinking water, and power generation, which lead to the consumption of water resources, and artificial impoundment increases water resources. From Figure 2a, the surface area and water level during the period of 2012–2013 both declined without a seasonal effect, possibly as a result of human activities (such as power generation). The surface area and water level both increased in 2014 because of artificial impoundment.

Figure 2b shows a good linear correlation between the changes in the DJKR area and the water level, with an R^2^ value of 0.99. Once an optimal relationship between the changes in area and water level is determined, it can be used to fill in and predict missing data on the area or water level, thus generating a longer and more efficient time series that can be used to verify the results from the GRACE data. This approach has useful applications in water resource management, flood control, and environmental monitoring of the MRSNWDP.

### 3.2. TWSA in the DJKR from the ILMM

#### 3.2.1. Results of the Scale Factor and Leakage

The SFM depends on the hydrological model and the smoothing method, but the DVM is independent of the hydrological model and only depends on the optimal average kernel. Here, the scale factors were obtained by different hydrological models and smoothing methods from the SFM and different smoothing methods from the DVM (Table 1).

As shown in Table 1, the scale factors are greater when the smoothed radius increases, which shows that more signals are attenuated for larger smoothed radii. The scale factors from different hydrological models are basically consistent with each other. The scale factors from the WGHM and CLM4.0 are larger than those from the CPC and GLDAS. The scale factor from the smoothed LMM is smaller than that from the 300-km Gauss filter by using the SFM; thus, the LMM can retain a more effective signal than that from the 300-km Gauss filter.

The scale factor results from the DVM show that as the smoothed radius increases, the scale factors also quickly increase. According to Equation (7), the scale factors largely depend on the study area. When the area is smaller, more and quicker signals are attenuated. The scale factor determined by using the DVM with a smoothed 200-km Gauss and the scale factor from the LMM are consistent with each other and closer to the results of the SFM than different hydrological models. This result shows that the key to scale factors from the DVM is to establish an approach that obtains the optimal regional average kernel, and the optimal kernel based on the LMM method can effectively preserve the signal and improve the spatial resolution.

**Figure 3:**
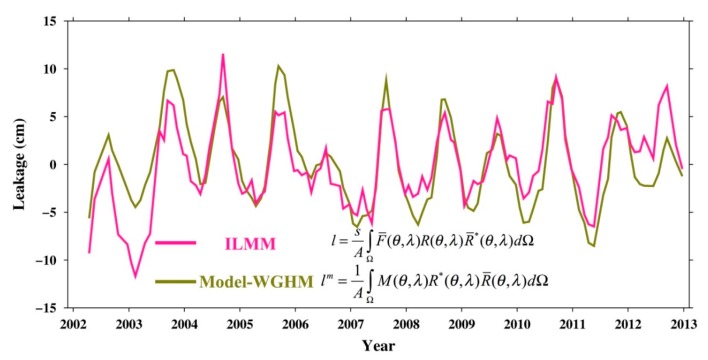
The leakage of the DJKR according to different methods.

The red line in Figure 3 represents the results of the DJKR leakage obtained from the LMM and Equation (13), and the other line represents data from the SFM (Equation (3)) with the smoothed 300-km Gauss and WGHM model. As shown in Figure 3, because the area of the DJKR is small, the leakage error is large. Therefore, correcting the signal leakage is crucial for determining the TWSA in small-scale basins.

#### 3.2.2. TWSA in the DJKR from Different Methods

**Figure 4:**
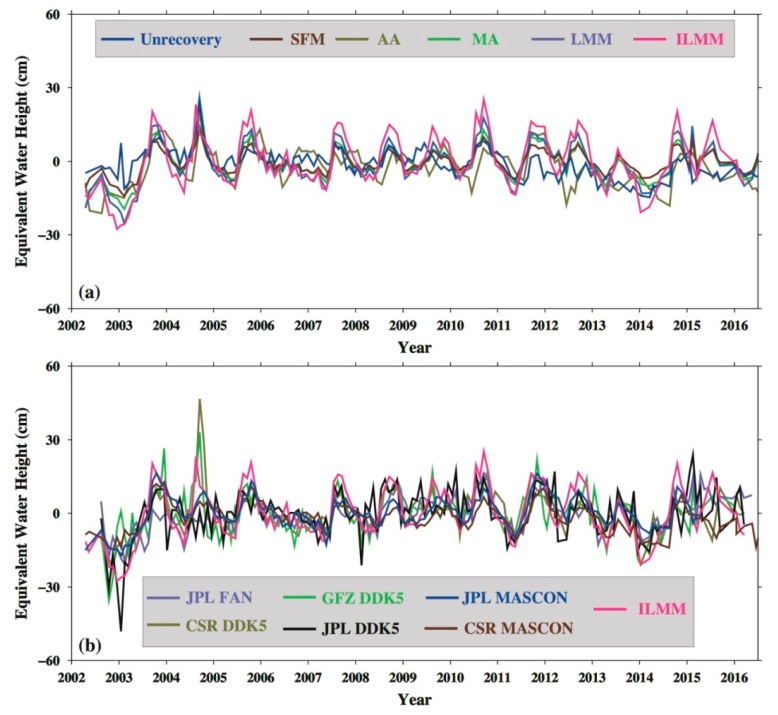
(**a**) Terrestrial water storage anomalies (TWSA) in the DJKR according to different methods and (**b**) different data. The ‘Unrecovery’ designates TWSA without restoring signals. The SFM, additive approach (AA), multiplicative approach (MA), Lagrange multiplier method (LMM), and improved Lagrange multiplier method (ILMM) are the different methods described in Section 2.3.3.

Figure 4 shows the time series of the TWSA in the DJKR according to different data sources and different post-processing methods. The results in Figure 4b are from different filtering methods (such as the FAN and DDK5 filters) by applying different data (such as CSR, JPL, and GFZ) and different MASCON products. As shown in Figure 4, the amplitude is slightly different, but the periodic signals are basically consistent with each other. The periodic signals of the TWSA from the ILMM are more obvious than those from other methods. The difference in the TWSA amplitude between January 2014 and November 2014 according to the ILMM is about 30 cm, which is the largest of the results of different methods.

### 3.3. DJKR Water Level and Storage Changes from GRACE, Altimetry, and Hydroclimatic Data

**Figure 5:**
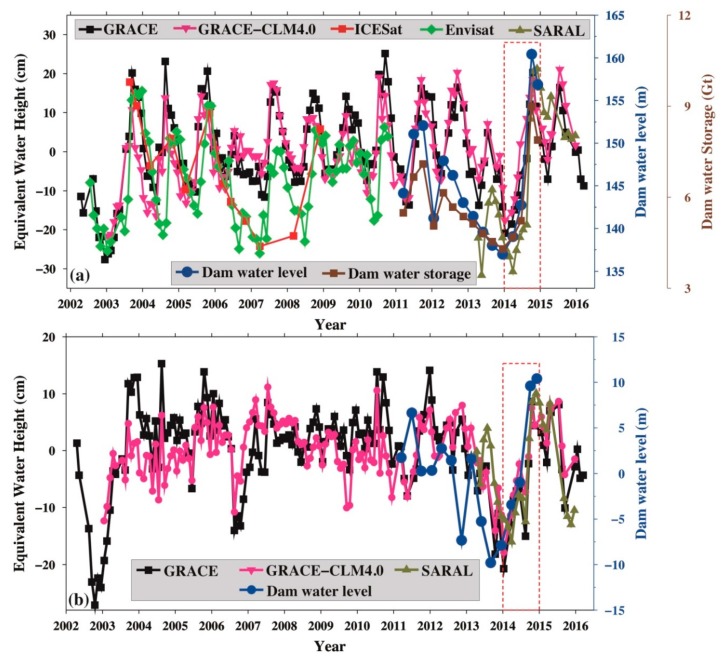
(**a**) Changes in the water level and storage from multi-source altimetry missions, in situ (DJKD), Gravity Recovery and Climate Experiment (GRACE), and CLM4.0 data; (**b**) the non-seasonal signal from GRACE, SARAL/Altika altimetry, and in situ (DJKD) water-level change.

As shown in Figure 5a, the periodic signals between water levels from the altimetry mission (ICESat, ENVISAT, and SARAL/Altika), TWSA from in situ observations of changes in the water level and area, and GRACE-TWSA agree well with each other. Figure 5a reveals that the 2006–2009 water-level data from ICESat and ENVISAT are in good agreement. The objective of the ICESat mission was to monitor mass changes in the ice sheets at the poles, which led to sparse laser footprints at middle and low latitudes. The signals of the water level from the ICESat mission were not well captured, especially between 2007–2008 (Figure 5a). Between 2012–2014, the water level declined as a result of human activities, such as power generation. This can be immediately captured by in situ data. Human activities cannot be reflected in real time because of the limitation of GRACE spatiotemporal resolution. Therefore, there are some peak TWSA values and human-induced TWSA, but no signals are detected in the in situ water level. The amplitudes between GRACE-TWSA and the water storage based on water level change and area data are slightly different.

The signals for the annual, semi-annual, and trend were removed by the least-squares fitting method, and the residual is called the non-seasonal signal [15], which is shown in Figure 5b. Figure 5b shows the non-seasonal signal of GRACE-TWSA, human-induced TWSA, the SARAL/Altika altimetry, and in situ (DJKD) water-level changes. From Figure 5a, the human-induced TWSA are weaker than those from GRACE-TWSA before 2011, but they were the same after 2011. The non-seasonal signal of GRACE-TWSA and human-induced TWSA from Figure 5b show the same results, indicating that the TWSA in the DJKR were influenced mostly by human activities after 2011 [50].

Figure 5 also shows a steep water level increase due to the impoundment in 2014, which is detected in the GRACE data and Altika altimetry data. Moreover, they agree well with the in situ data. According to the above results, the DJKR impoundment signal in 2014 was captured by GRACE, altimetry missions, and in situ data, indicating that GRACE and altimetry can be effectively used to monitor the surface water change in a small reservoir.

**Figure 6:**
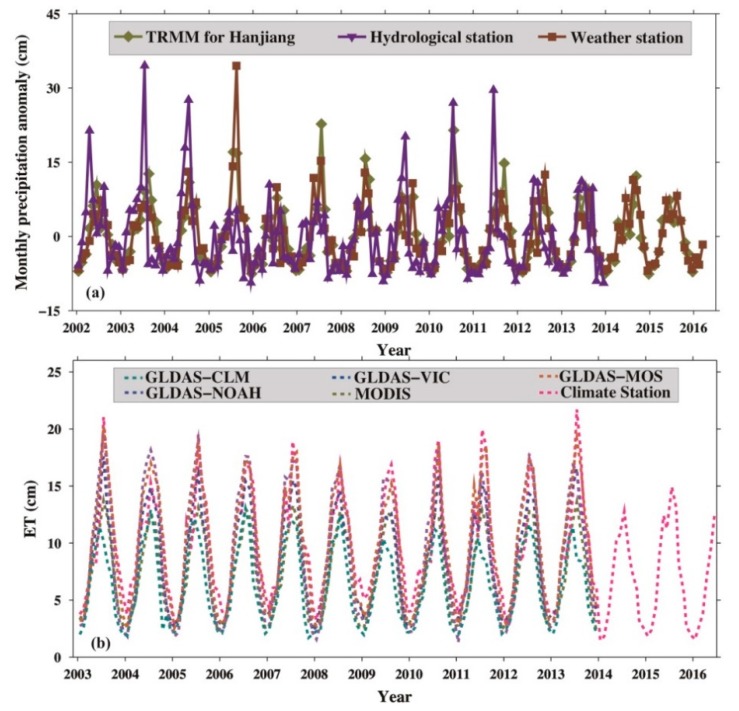
(**a**) Change in precipitation in the DJKR from the Tropical Rainfall Measuring Mission (TRMM), hydrological station, and weather station; (**b**) evapotranspiration (ET) in the DJKR from the four GLDAS models, Moderate Resolution Imaging Spectroradiometer (MODIS), and weather station.

Figure 6a shows the change in precipitation in the Hanjiang River basin (HRB) from the TRMM, the Xiantao hydrological control station, and the Danjiangkou city weather station. The Xiantao hydrological control station exhibits hydrological information for the entire HRB. The results show that the HRB precipitation data from the TRMM and the Xiantao hydrological control station are basically the same, and the correlation coefficients between them are over 0.8. They agree well with the precipitation data from the Danjiangkou city weather station, except for amplitude differences in the summer; thus, the data from the TRMM are sufficient to quantify the HRB precipitation. From the strong relationship between the monthly precipitations data from the TRMM and the Danjiangkou city weather station (e.g., high correlation coefficients), the rainfall situation for the entire HRB and DJKR are basically the same. Figure 6b shows the evapotranspiration in the Hanjiang River basin and the DJKR from the four GLDAS models, MODIS, and the Danjiangkou city weather station, and it indicates that evapotranspiration has a large uncertainty. As shown in Figure 6, the water in the HRB was sufficient. Precipitation and evapotranspiration were steady, without heavy rainfall and/or low evaporation in 2014.

**Figure 7:**
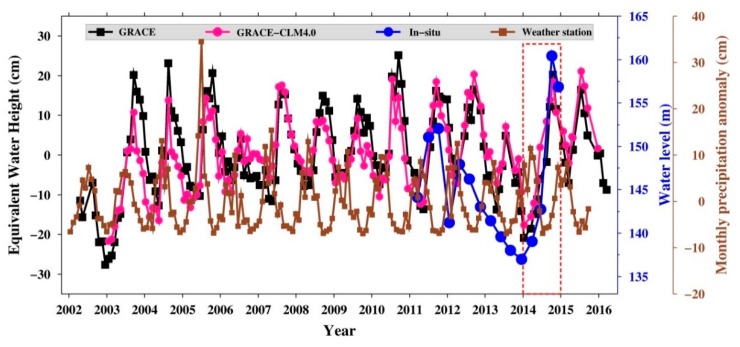
Comparison of the changes in water storage, precipitation, and water level.

The TWSA from GRACE and CLM4.0 data, water-level change from in situ data, and precipitation from the Danjiangkou city weather station are all compared in Figure 7. A time lag between precipitation and GRACE-TWSA is revealed in this figure; in other words, when precipitation increases or decreases, GRACE-TWSA responds by increasing or decreasing after several months. The maximum correlation of 0.62 between precipitation and GRACE-TWSA is at a two-month lag (Figure S1). The results also show that the HRB precipitation was steady without heavy rainfall in 2014, but the human-driven surface water storage change shows a steep increase. Therefore, the signal for the steep water level increase is due to impoundment by humans, which can be verified by GRACE, altimetry, and in situ data.

### 3.4. Fingerprints of the MRSNWDP in the DJKR

Impoundment would be expected to affect the water storage in the HRB and DJKR. In order to investigate the fingerprints in the DJKR, an analysis was carried out on the spatiotemporal changes in precipitation from the TRMM, TSWSA from GRACE and GLDAS data, as well as HSWSA from GRACE and CLM4.0 data.

**Figure 8:**
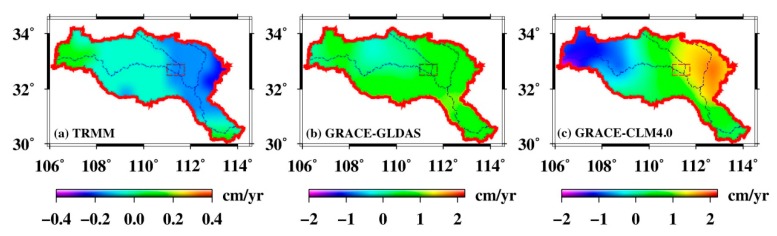
(**a**) Trends of the changes in precipitation from the TRMM, (**b**) total surface water storage from GRACE-GLDAS, and (**c**) human-induced surface water storage from GRACE-CLM4.0 during the period of 2003–2015.

As shown in Figure 8, the water storage increased in the upper HRB, but decreased in the lower HRB. This agrees with the goal of the SNWDP, which is the diversion of water to the north from the south in China to address the increasing reduction of water in northern China. The TSWSA was fairly steady, without a significant surplus or shortage, but the HSWSA decreased in the upper HRB and increased in the lower HRB.

**Figure 9:**
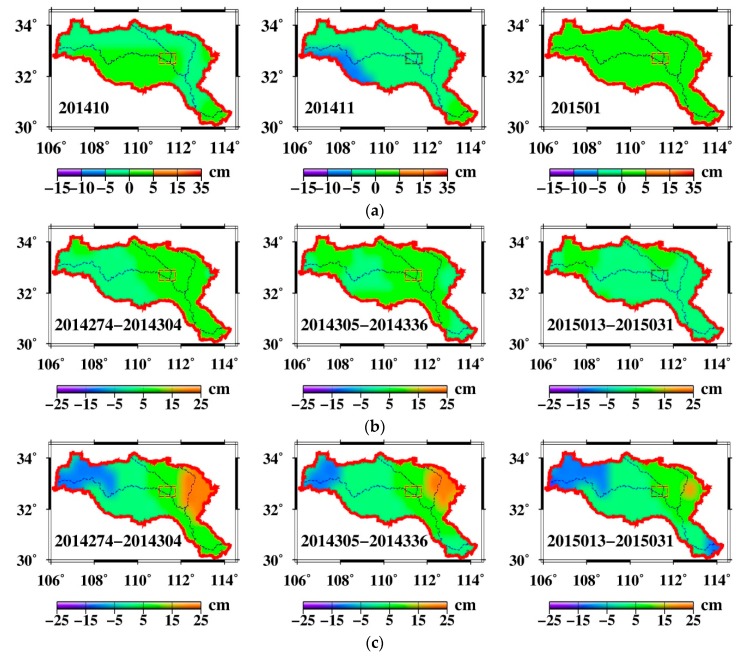
(**a**) Changes in the precipitation, (**b**) total surface water storage, and (**c**) human-induced surface water storage in October 2014, November 2014, and January 2015.

To analyze the water diversion fingerprints due to the DJKR impoundment, each month’s precipitation, TSWSA, and HSWSA between October 2013 and December 2015 were obtained (Figure S2). The precipitation change and TSWSA were basically steady and water sufficient; this observation indicates that the TSWSA were mainly influenced by the rainfall in this period (Figure S2). However, regardless of the precipitation change, the HSWSA obviously decreased in the upper HRB and increased in the DJKR and east of it, especially between October 2014 and January 2015 (Figure 9). The water diversion fingerprints (Figure S2) show the progress of water resources in the upper HRB as water is impounded to the DJKR. The impoundment caused water in the upper region to decrease and water from the DJKR and the east of it to increase. The above results reveal that the water diversion fingerprints due to the DJKR impoundment can be captured by the GRACE mission.

## 4. Discussion

### 4.1. Verification TWSA from ILMM

In this study, the results show that ILMM can be used to effectively infer the TWSA in small basins (such as DJKR); thus, with the ILMM, there is a high potential for the full exploitation of GRACE data for hydrology research. GRACE-TWSA of the DJKR should be verified by water balance equations [20]. Unfortunately, we do not have in situ runoff data for the DJKR. Moreover, evapotranspiration is difficult to estimate and has a large uncertainty (Figure 6b) [69]. Therefore, the TWSA obtained from ILMM are compared with different methods, and the result shows that the TWSA from the different methods agree with each other, but the periods and amplitudes from the ILMM are the most pronounced. The impoundment signal in 2014 was only detected by using the ILMM (Figure 4). Additionally, five methods are used to extract the TWSA from GRACE data, which can also be used to verify the accuracy of different hydrological models in different regions.

Additionally, there are no effective methods that can fully verify the results of GRACE, and this is one of the key limitations in the application of GRACE. Presently, the most effective method for verifying the results from GRACE is comparing GRACE data with in situ hydrological station data. Although GRACE’s main signal can be considered hydrological data after deducing other signals from the background model, the TWSA from GRACE represent the total surface mass change, which is different from hydrological data. In addition, there are errors in the background model and hydrological data, which can also affect the results. Therefore, it is still necessary to further study an effective method in order to verify the accuracy of the TWSA detected from GRACE data.

### 4.2. Combination of In Situ, GRACE, and Altimetry Data to Manage Reservoir Water Resources

Changes in the water level and storage in artificial reservoirs are important components of the water cycle and water resources. With the increasing use of reservoirs, the annual and seasonal flow will change, and water cycles will be affected. There is an urgent need to study reservoir water resource management. Compared with satellite remote sensing technology, in situ data can provide observational data with higher precision, and are more appropriate for monitoring an individual water body of importance. Although the satellite is important for monitoring water storage changes, its advantage is its consecutive data and ability to cover large areas. Therefore, the potential capacity of satellites (such as GRACE and altimetry missions) to monitor water resources in small-scale basins should be established. In situ and remote sensing data can be combined to manage global and regional water resources.

Many studies have indicated that GRACE and altimetry can be used to detect and manage water resources, especially in remote regions and in transboundary countries [7]. Here, we also confirm that the combination of GRACE remote sensing, altimetry, and model data can provide another effective method for understanding the hydrological processes in small reservoirs.

The combination of satellite gravimetry and multi-source altimetry provide a unique tool for global water resource management. With the development of space technology, regional and global hydrological models that are developed by means of space-based observations will aid in the management of water resources [20] and disrupt the deadlock caused by unavailable data and management opacity. Combining hydrological remote sensing observational data, hydrological modeling, and existing high-precision hydrological station observational data can provide a wide range of high-precision freshwater maps for a particular region or the entire world. This kind of scientific research is essential for more efficient, sustainable, and cross-boundary cooperative water resource management.

## 5. Conclusions

The surface water in rivers, lakes, and reservoirs is an important part of the environment and a key strategic resource for human development. Artificial reservoirs play an important role in water supply and flood control, and may become more critical with the increasing frequency and intensity of extreme weather events. However, few studies have focused on small artificial reservoirs. Here, we propose the ILMM to improve the potential of the GRACE mission for detecting the TWSA in the DJKR. The water diversion fingerprints due to DJKR impoundment are quantified and analyzed by using GRACE, multi-source altimetry, Landsat, land surface model, precipitation, and in situ data. The main findings are as follows:

(1) Changes in the water level and area in the DJKR show a good linear correlation. The relationship between the changes in area and changes in the water level is determined, and it can be used to fill in and predict missing data, verify the TWSA from GRACE data, and benefit water resource management.
(2) The ILMM can improve the spatial resolution and enable the use of GRACE data to detect the TWSA in small-scale basins (such as DJKR).
(3) GRACE and altimetry missions can be effectively used to monitor human-induced surface water changes, such as the impoundment of small artificial reservoirs.
(4) The precipitation change and TSWSA in the HRB are basically steady and water sufficient. According to GRACE and CLM4.0 data, regardless of the precipitation changes, HSWSA obviously decreased in the upper HRB and increased in the DJKR and to the east of it, which indicates that these are the human-induced TWSA. The GRACE mission can capture the phenomenon, i.e., the water diversion fingerprints due to the DJKR impoundment.

The GRACE mission has made significant and unique contributions to Earth science. This mission ended in October 2017. The GRACE Follow-On (GFO) mission was launched in May 2018, and the SWARM constellation [70] has the potential to determine the time-variable gravity field and the TWSA [71]. Therefore, we can combine GRACE, GFO, and SWARM data to detect Earth’s mass transport. Additionally, the altimetry missions CryoSat-2, ICESat-2, and future missions (Water Cycle Observation Mission (WCOM) and Water and Ocean Topography (SWOT)) [72] can be used to monitor water resources, and all of these missions have the potential to complement or replace in situ datasets for global or regional water resource management.

## Figures and Tables

**Table 1:** Scale factors from different methods. CLM4.0: Community Land Model, CPC: Climate Prediction Center, DVM: scale factor independent of the hydrological model proposed by Dutt Vishwakarma et al. [65]; GLDAS: Global Land Data Assimilation System, LMM: Lagrange multiplier method; SFM: scale factor method, WaterGAP Global Hydrology Model.

Scale Methods		Smoothed Methods				
		Gauss Smooth Radius (Units: km)				LMM
		200	300	400	500	
DVM		3.67	4.40	6.59	9.56	2.48
SFM with different hydrological models	CPC	1.36	1.68	1.98	2.20	1.63
	GLDAS	1.39	1.70	1.95	2.11	1.66
	WGHM	1.41	1.72	2.01	2.25	1.70
	CLM4.0	1.42	1.71	2.02	2.23	1.68

## Supplementary Materials

The following are available online at https://www.mdpi.com/1424-8220/19/16/3510/s1, the cross-correlation coefficient between TWSA and precipitation (Figure S1), and changes in the precipitation (left), total surface water storage (middle), and human-induced surface water storage (right) between October 2003 and December 2015 (Figure S2).

## Abbreviations

AA	Additive approach
CLM	Community Land Model
CPC	Climate Prediction Center
DJKR	Danjiangkou Reservoir
DVM	Dutt Vishwakarma et al. [65] Method
GLDAS	Global Land Data Assimilation System
GRACE	Gravity Recovery and Climate Experiment
GRACE-TWSA	Terrestrial Water Storage Anomaly from GRACE data
GWSA	GroundWater Storage Anomaly
HRB	Hanjiang River Basin
HSWSA	Human-induced Surface Water Storage Anomaly
ILMM	Improved Lagrange Multiplier Method
LMM	Lagrange Multiplier Method
MA	Multiplicative Approach
SFM	Scale factor Method
SMA	Soil Moisture Anomaly
SWSA	Surface Water Storage Anomaly
TRMM	Tropical Rainfall Measuring Mission
TSWSA	Total Surface Water Storage Anomaly
TWSA	Terrestrial Water Storage Anomalies
WGHM	WaterGAP Global Hydrology Model

## Appendix A. Lagrange Multiplier Method

Wahr et al. [40] proposed a simple Gaussian smoothing method to determine the terrestrial water storage anomalies (TWSA); however, the method was unable to isolate a specific basin. Additional technology is required to estimate basin TWSA by using GRACE data.

The vertical integration of water storage in any average region can be written as follows [66]:

(A1) $${\bar{TWSA}}_{region}=\frac{1}{\Omega_{region}}\int TWSA(\theta ,\lambda )\vartheta (\theta ,\lambda )d\Omega$$

where *λ* is the geocenter longitude, and *θ* is the geocenter latitude. The accurate average kernel ϑ(θ,λ) is the function of the shape of the described regional (such as a river basin):

(A2) $$\vartheta (\theta ,\lambda )=\{\begin{array}{cc} 0 & outside\;\mathrm{the}\;\mathrm{basin}\\ 1 & inside\;\mathrm{the}\;\mathrm{basin} \end{array}$$

where dΩ=sinλdλdθ is the solid angle element, and the integration in a given spherical domain $\Omega_{region}$ (spherical area) is ϑ(θ,λ). From Equations (1) and (A1), we can obtain

(A3) $${\bar{TWSA}}_{region}=\frac{a\rho_{ave}}{3\Omega_{region}}\sum_{l=0}^{\infty }\sum_{m=0}^{l}\frac{\left(2l+1\right)}{\left(1+k_{l}\right)}(\vartheta_{lm}^{c}\Delta C_{lm}+\vartheta_{lm}^{s}\Delta S_{lm})$$

where *a* is the semi-major axis of the reference ellipsoid; $\rho_{ave}$ is the average earth density (5517 kg/m^3^); $k_{l}$ is the Love number [59]; l,m are the degree and order; $\Delta C_{lm}$, $\Delta S_{lm}$ are the residual spherical harmonic coefficient after subtracted the long-term mean of the Earth gravity field; $\vartheta_{lm}^{c}$ and $\vartheta_{lm}^{s}$ are the spherical harmonic coefficients of ϑ(θ,λ):

(A4) $$\vartheta (\theta ,\lambda )=\frac{1}{4\pi }\sum_{l=0}^{\infty }\sum_{m=0}^{l}{\tilde{P}}_{lm}(\cos\theta )\{\vartheta_{lm}^{c}\cos m\lambda +\vartheta_{lm}^{s}\sin m\lambda \}$$

(A5) $$\left\{\begin{array}{c} \vartheta_{lm}^{c}\\ \vartheta_{lm}^{s} \end{array}\right\}=\int \vartheta (\theta ,\lambda ){\tilde{P}}_{lm}(\cos\theta )\left\{\begin{array}{c} \cos m\lambda \\ \sin m\lambda \end{array}\right\}d\Omega$$

Using $\bar{W}(\theta ,\lambda )$ to replace the accurate average kernel ϑ(θ,λ) in Equation (A1),

(A6) $${\tilde{TWSA}}_{region}=\frac{1}{\Omega_{region}}\int TWSA(\theta ,\lambda )\bar{W}(\theta ,\lambda )d\Omega$$

where ${\tilde{TWSA}}_{region}$ is the approximate regional average, and $\bar{W}$ can be expanded to obtain the following:

(A7) $$\bar{W}(\theta ,\lambda )=\frac{1}{4\pi }\sum_{l=0}^{l_{trnc}}\sum_{m=0}^{l}{\tilde{P}}_{lm}(\cos\theta )\{W_{lm}^{c}\cos m\lambda +W_{lm}^{s}\sin m\lambda \}$$

The approximate regional average can be approximately expressed by using the spherical harmonic coefficients:

(A8) $${\tilde{TWSA}}_{region}=\sum_{l,m}\frac{K_{l}}{\Omega_{region}}(W_{lm}^{c}\Delta C_{lm}+W_{lm}^{s}\Delta S_{lm})$$

where $K_{l}=\frac{a\rho_{E}}{3}\frac{\left(2l+1\right)}{\left(1+k_{l}\right)}$.

The variance of the corresponding satellite measurement error is

(A9) $$\mathrm{var}(\varepsilon_{sat})=\frac{1}{\Omega_{region}^{2}}\sum_{l,m}\frac{K_{l}^{2}{\;B}_{l}^{2}}{2l+1}[W_{lm}^{c}{}^{2}+W_{lm}^{s}{}^{2}])$$

where $B_{l}^{2}=\frac{1}{n}\sum_{i=1}^{n}\sum_{m=0}^{l}{\left[{\left(\Delta \delta_{lm}^{c}\right)}^{2}+\Delta \delta_{lm}^{s}\right)}^{2}]$, $\left(\Delta \delta_{lm}^{c},\Delta \delta_{lm}^{s}\right)$ is the nominal error of GRACE data [34].

The Lagrange multiplier method fixes one type of error (satellite or leakage errors) to a specific value and minimizes the other type of error (leakage or satellite errors). A type of signal leakage is defined as the ratio of the variance of accurate and approximate average kernel difference to the variance of the accurate average kernel:

(A10) $$\begin{array}{ll} \mathrm{var}(\varepsilon_{lkg}) & =\frac{{\int \left[\bar{W}(\theta ,\lambda )-\vartheta (\theta ,\lambda )\right]}^{2}d\Omega }{{\int \left[\vartheta (\theta ,\lambda )\right]}^{2}d\Omega }\\ & =\frac{1}{4\pi \Omega_{region}}\sum_{l,m}\left[{\left(W_{lm}^{c}-\vartheta_{lm}^{c}\right)}^{2}+{\left(W_{lm}^{s}-\vartheta_{lm}^{s}\right)}^{2}\right] \end{array}$$

Let $\delta^{2}$ be the variance of satellite measurement error (Equation (A9)) and $\Delta^{2}=\delta^{2}\Omega_{region}^{2}$, which are used to establish the Lagrange multiplier equation (Equation (A11)). $W_{lm}^{c}$, $W_{lm}^{s}$, and *λ* can be determined through the minimum value of Equation (A11):

(A11) $$\begin{array}{l} \xi =\sum_{l,m}\left\{{\left[W_{lm}^{c}-\vartheta_{lm}^{c}\right]}^{2}+{\left[W_{lm}^{s}-\vartheta_{lm}^{s}\right]}^{2}\right\}\\ \;+\lambda \left\{\sum_{l,m}\frac{K_{l}^{2}B_{l}^{2}}{2l+1}[W_{lm}^{c}{}^{2}+W_{lm}^{s}{}^{2}]-\Delta^{2}\right\} \end{array}$$

We seek the partial derivative of $W_{lm}^{c}$, $W_{lm}^{s}$, and *λ* of ξ and set them to zero to obtain

(A12) $$\left\{\begin{array}{c} W_{lm}^{c}\\ W_{lm}^{s} \end{array}\right\}={\left[1+\lambda \frac{K_{l}^{2}B_{l}^{2}}{2l+1}\right]}^{-1}\left\{\begin{array}{c} \vartheta_{lm}^{c}\\ \vartheta_{lm}^{s} \end{array}\right\}$$

(A13) $$\sum_{l,m}\frac{K_{l}^{2}B_{l}^{2}}{2l+1}[W_{lm}^{c}{}^{2}+W_{lm}^{s}{}^{2}]=\Delta^{2}$$

Combining Equations (A12) and (A13), we obtain

(A14) $$\sum_{l,m}\frac{K_{l}^{2}B_{l}^{2}}{2l+1}\frac{\vartheta_{lm}^{c}{}^{2}+\vartheta_{lm}^{s}{}^{2}}{{\left[1+\lambda \frac{K_{l}^{2}B_{l}^{2}}{2l+1}\right]}^{2}}=\Delta^{2}$$

When *λ* is obtained from Equation (A14), we can plug it into Equation (A12) to obtain $W_{lm}^{c}$ and $W_{lm}^{s}$. Finally, we put them into the Equation (A8), and TWSA can be inferred. This is the Lagrange multiplier method, which is the fixed satellite measurement error to minimize the signal leakage error.
