# Supplementary material for: Detecting Water Diversion Fingerprints in the Danjiangkou Reservoir from Satellite Gravimetry and Altimetry Data
Source: Sensors (Basel). 2019 Aug 10;19(16):3510. doi: 10.3390/s19163510 (PMC6721160; doi:10.3390/s19163510)
Supplement: Supplementary file 1 [file sensors-19-03510-s001.pdf]

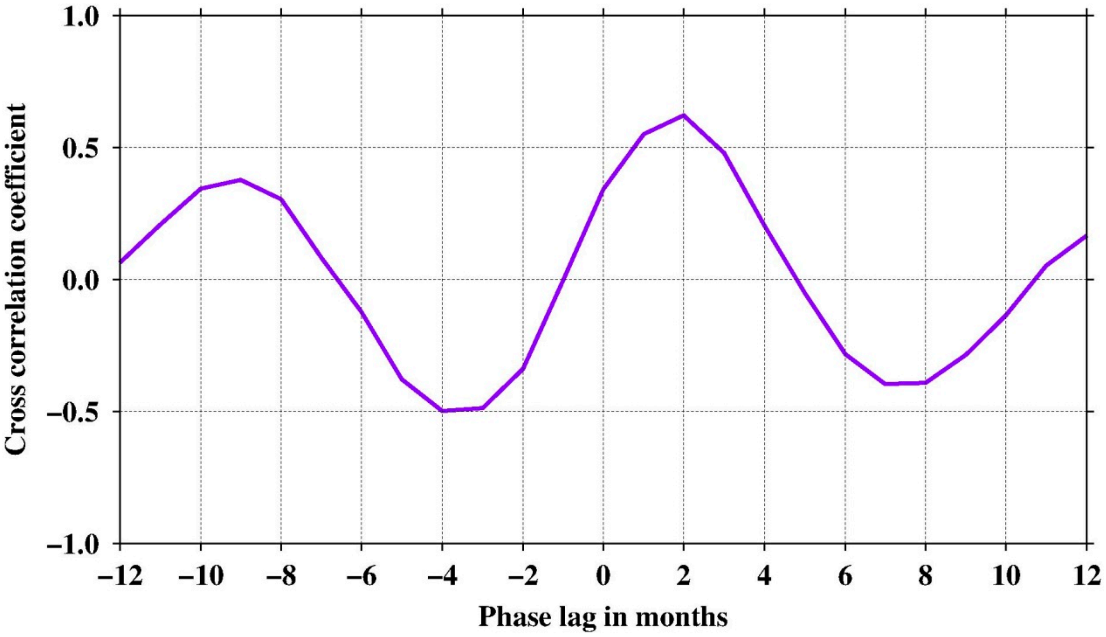

Figure S1. Cross-correlation coefficients between TWSA and precipitation.

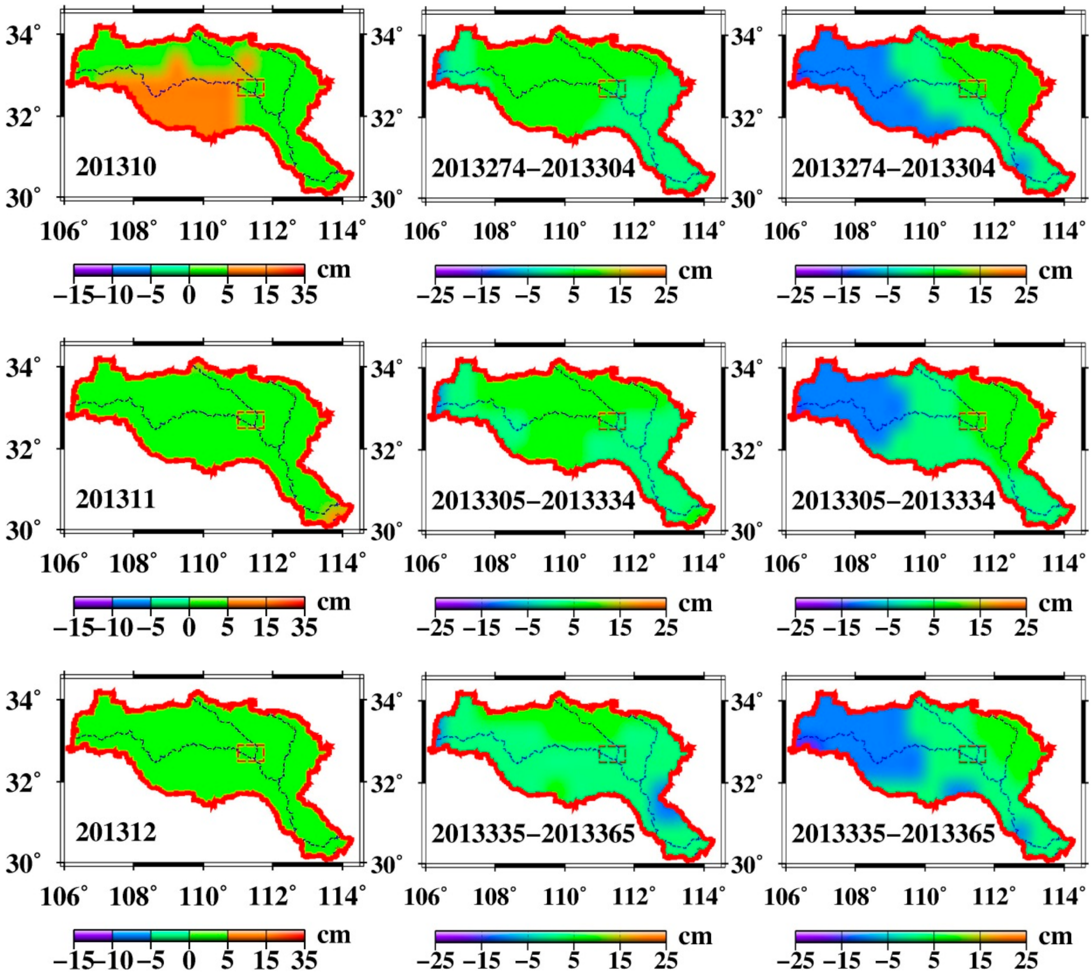

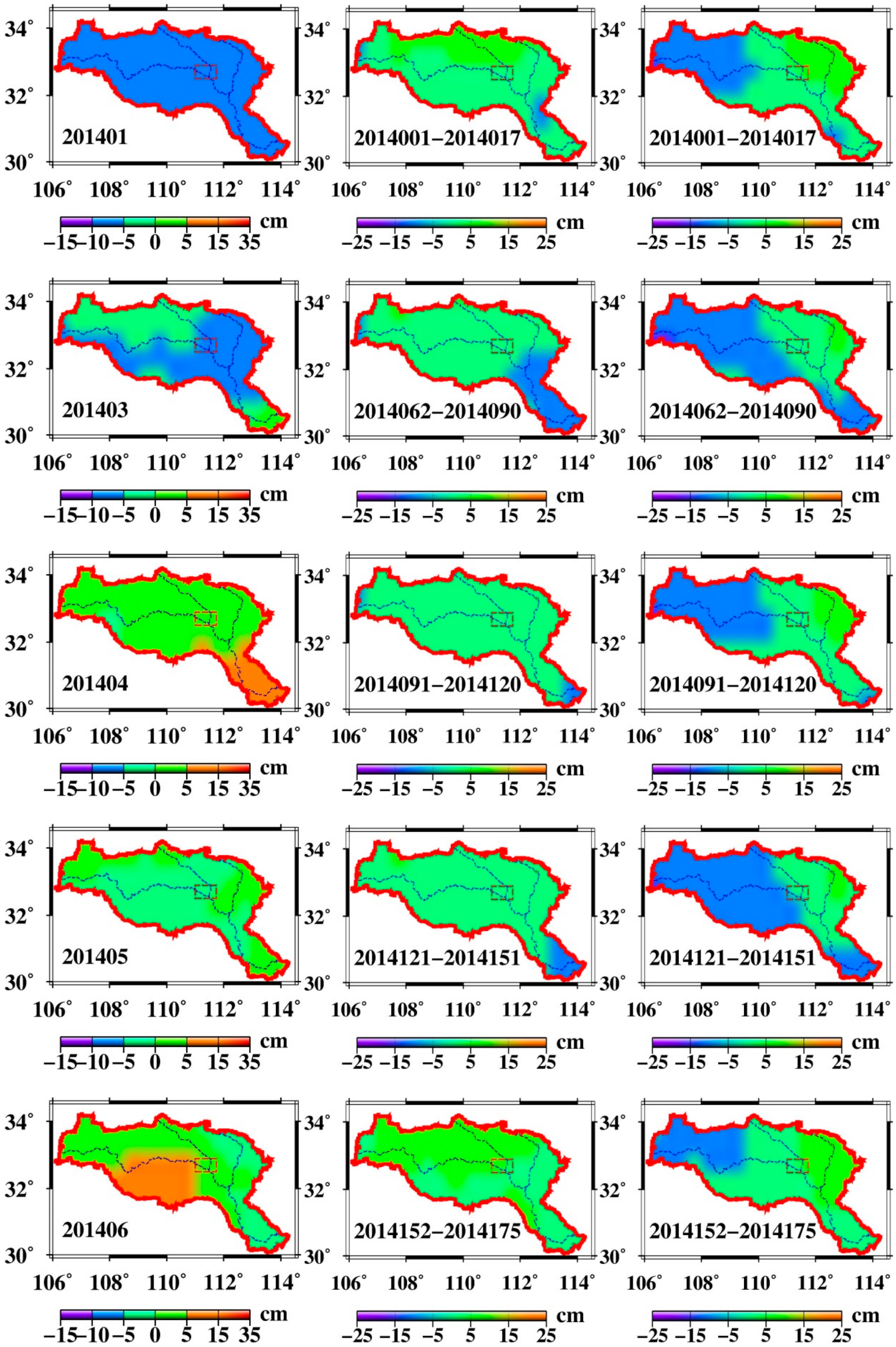
201401
2014001−2014017
2014001−2014017
201403
2014062−2014090
2014062−2014090
201404
2014091−2014120
2014091−2014120
201405
2014121−2014151
2014121−2014151
201406
2014152−2014175
2014152−2014175
30° 32° 34°
106° 108° 110° 112° 114°
cm
−15 −10 −5 0 5 15 35
−25 −15 −5 5 15 25

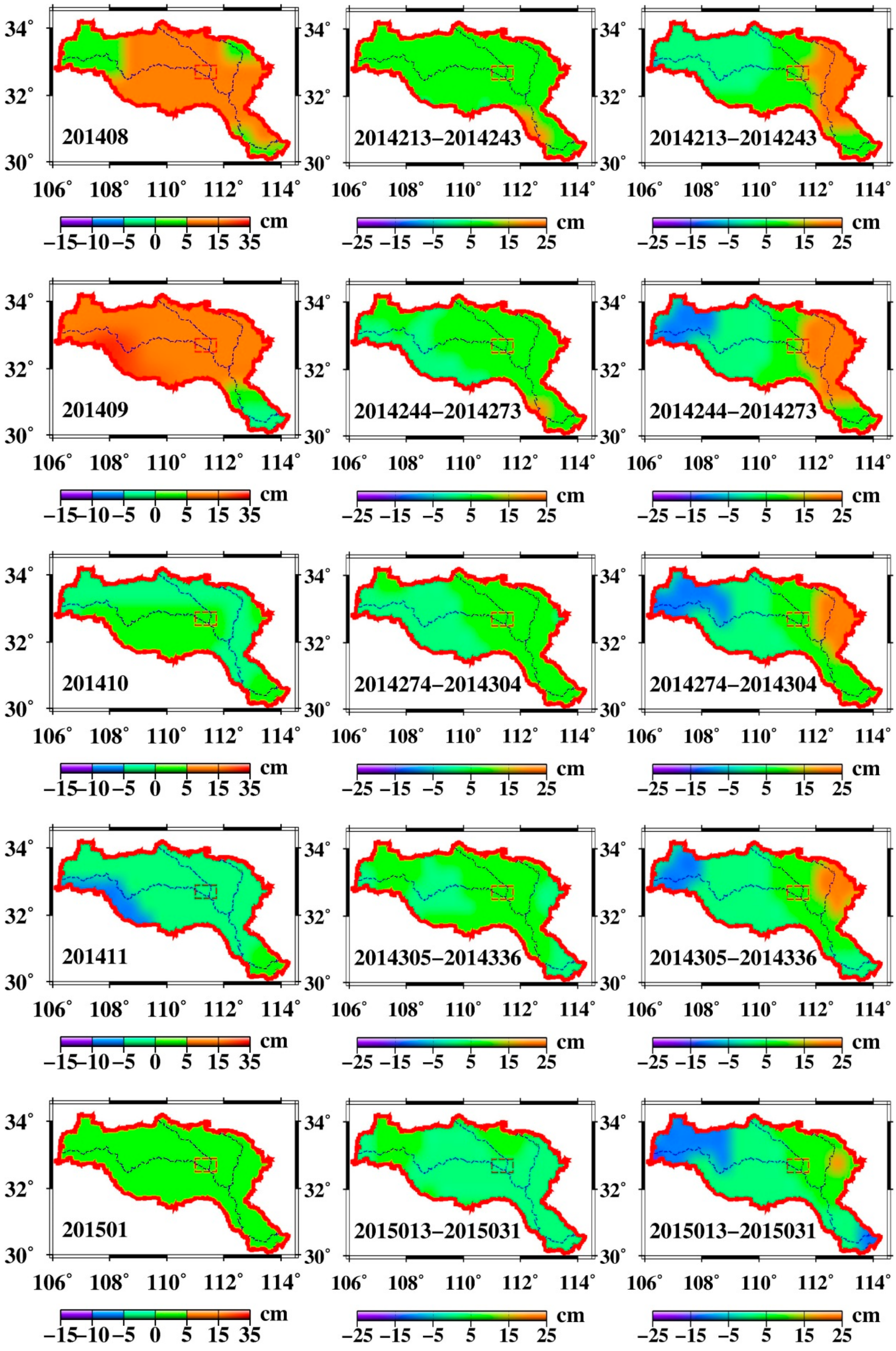
201408
2014213–2014243
2014213–2014243
201409
2014244–2014273
2014244–2014273
201410
2014274–2014304
2014274–2014304
201411
2014305–2014336
2014305–2014336
201501
2015013–2015031
2015013–2015031
30° 32° 34°
106° 108° 110° 112° 114°
−15 −10 −5 0 5 15 35 cm
−25 −15 −5 5 15 25 cm

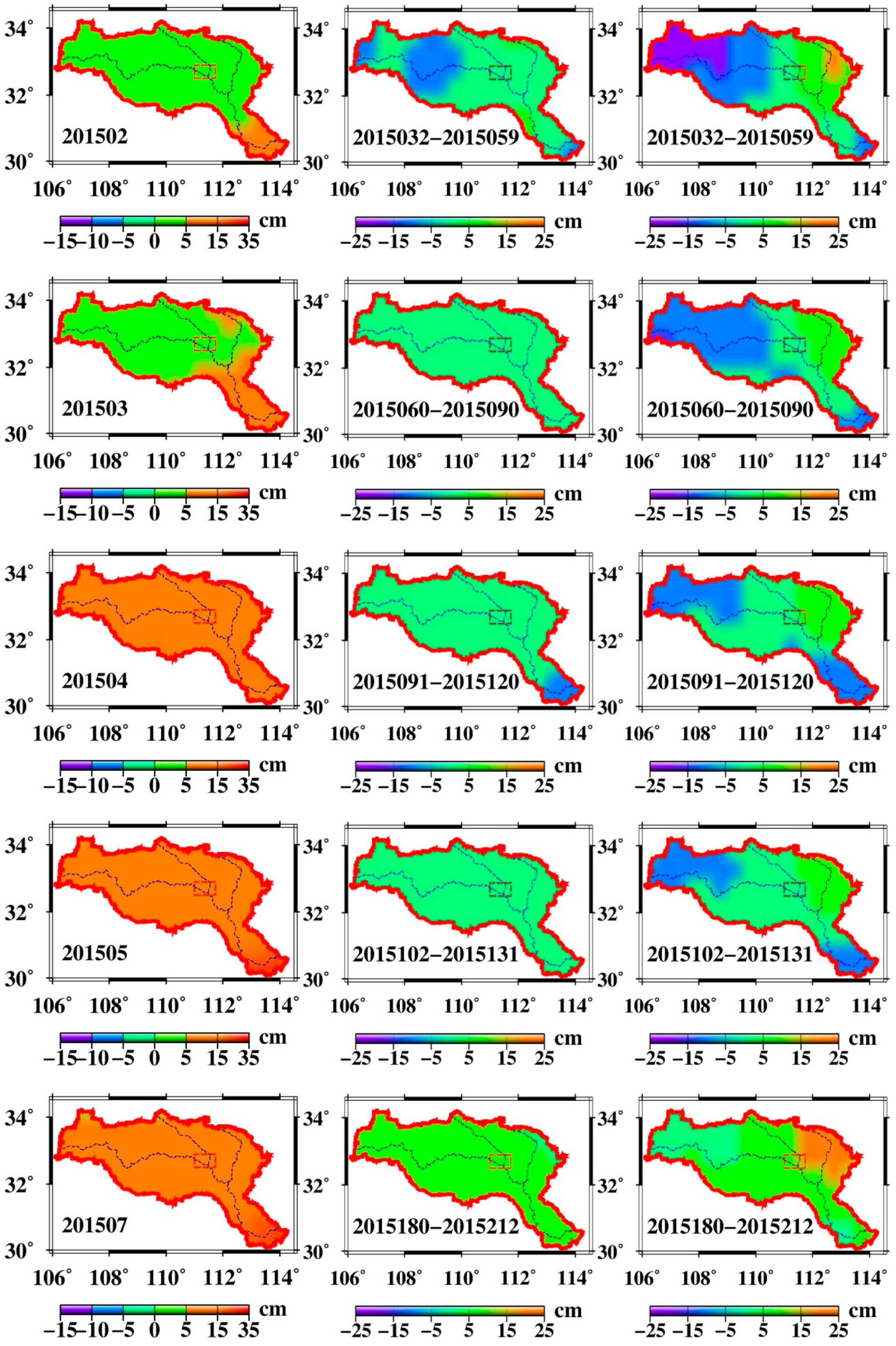
201502
2015032−2015059
2015032−2015059
201503
2015060−2015090
2015060−2015090
201504
2015091−2015120
2015091−2015120
201505
2015102−2015131
2015102−2015131
201507
2015180−2015212
2015180−2015212
106° 108° 110° 112° 114°
30° 32° 34°
−15 −10 −5 0 5 15 35 cm
−25 −15 −5 5 15 25 cm

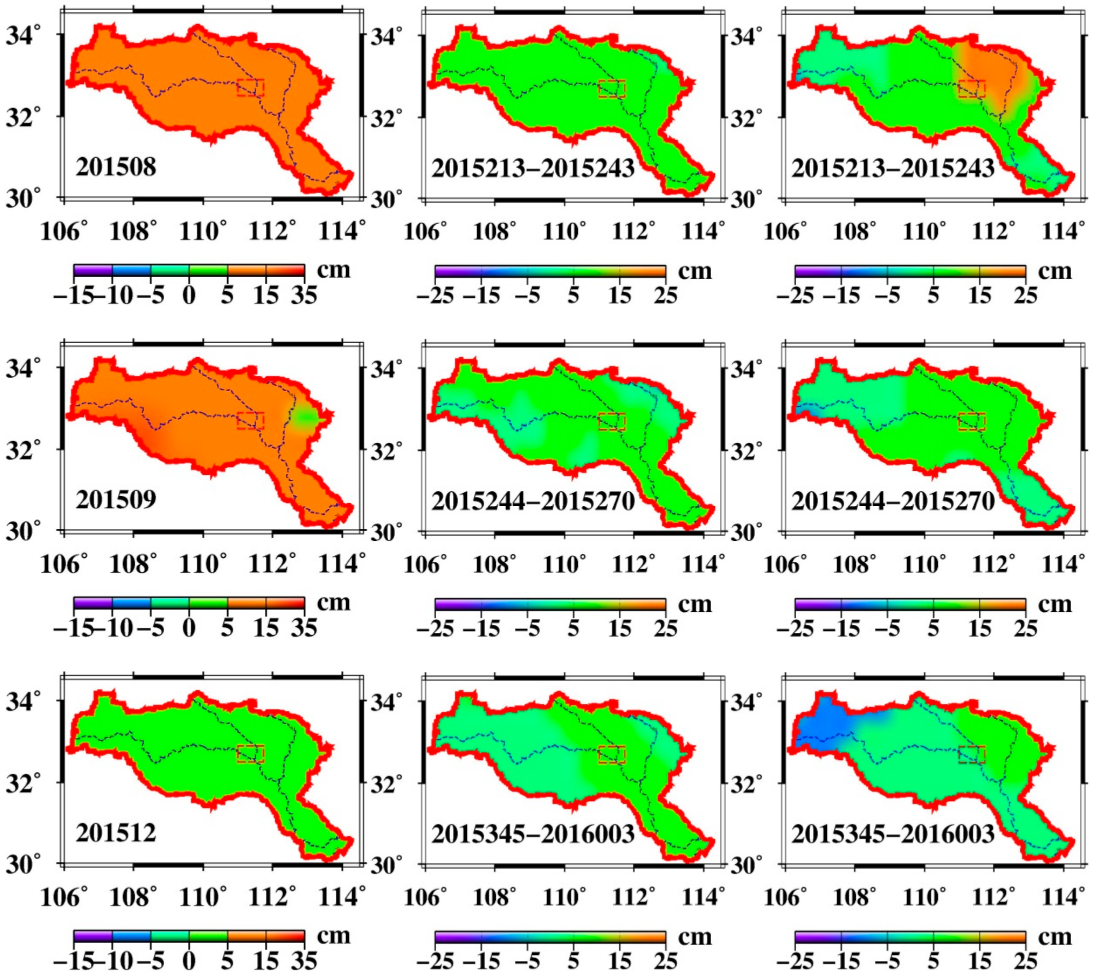

Figure S2. (**Left**) Changes in the precipitation, (**Middle**) total surface water storage and (**Right**) human-induced surface water storage between October 2013 and December 2015.
